# Heat Shock Protein-27, -60 and -90 expression in gastric cancer: association with clinicopathological variables and patient survival

**DOI:** 10.1186/1471-230X-9-14

**Published:** 2009-02-09

**Authors:** Constantinos Giaginis, Stella S Daskalopoulou, Stephanie Vgenopoulou, Ioannis Sfiniadakis, Gregorios Kouraklis, Stamatios E Theocharis

**Affiliations:** 1 Department of Forensic Medicine and Toxicology, Medical School, University of Athens, Athens, Greece; 2 2ndDepartment of Propedeutic Surgery, Medical School, University of Athens, Athens, Greece; 3 Department of Pathology, Naval Hospital, Athens, Greece

## Abstract

**Background:**

Heat shock proteins (HSPs) are ubiquitous, highly conserved proteins across all the species and play essential roles in maintaining protein stability within the cells under normal conditions, while preventing stress-induced cellular damage. HSPs were also overexpressed in various types of cancer, being associated with tumor cell proliferation, differentiation and apoptosis. The aim of the present study was to evaluate the clinical significance of HSP -27, -60, and -90 expression in gastric carcinoma.

**Methods:**

HSP -27, -60, and -90 proteins expression was assessed immunohistochemically in tumoral samples of 66 gastric adenocarcinoma patients and was statistically analyzed in relation to various clinicopathological characteristics, tumor proliferative capacity and patients' survival.

**Results:**

HSP-27, -60, -90 proteins were abundantly expressed in gastric adenocarcinoma cases examined. HSP-27 expression was significantly associated with tumor size (pT, P = 0.026), the presence of organ metastases (pM, P = 0.046) and pStage (P = 0.041), while HSP-27 staining intensity with nodal status (pN, P = 0.042). HSP-60 expression was significantly associated with patients' sex (P = 0.011), while HSP-60 staining intensity with patients' age (P = 0.027) and tumor histopathological grade (P = 0.031). HSP-90 expression was not associated with any of the clinicopathological parameters examined; however, HSP-90 staining intensity was significantly associated with tumor size (pT, P = 0.020). High HSP-90 expression was significantly associated with longer overall survival times in univariate analysis (log-rank test, P = 0.033), being also identified as an independent prognostic factor in multivariate analysis (P = 0.026).

**Conclusion:**

HSP-27, -60, and -90 were associated with certain clinicopathological parameters which are crucial for the management of gastric adenocarcinoma patient. HSP-90 expression may also be an independent prognostic indicator in gastric adenocarcinoma patients.

## 1. Background

Heat shock proteins (HSPs) are ubiquitous, highly conserved proteins across all species, which are strongly induced by heat shock and diverse environmental and physiopathological stresses [1,2]. HSPs constitute the products of several distinct genes commonly designated according to their mass [1-3]. Their principal function as molecular chaperones results in the maintenance of stability and delivery of other peptides, which are crucial for the protection of cellular integrity in normal and malignant cell growth. In absence of stress, HSPs form complexes with the heat shock transcription factors (HSFs), which remain in an inactive form [4]. During stress conditions, HSPs repress chaperones and link to misfolded proteins, which allows the activation of HSF through phosphorylation by protein kinases (PK), such as PKC and serine/threonine kinases [4-6]. Hence, HSF forms a homotrimeric structure in the cytosol and translocate to the nucleus, binding to heat shock elements (HSEs) in order for the transactivation of heat shock-inducible genes to be elicited [4-6].

Aside from their response to heat shock and chemical or physical stress stimuli, HSPs have been reported to be overexpressed in a wide range of human tumors including breast, endometrial, ovarian, colon, lung and prostate [7]. The expression of several HSPs has also been shown to correlate with tumor cell proliferation and differentiation, as well as apoptosis-related molecules in various types of cancer [7]. Currently, several drugs have been advanced in clinical studies rendering HSPs as emerging therapeutic targets in fighting cancer [4,7]. To this point of view, HSP-90 targeted drugs, such as 17AAG and SNX2112, are currently being advanced in order to selectively inhibit HSP-90 in tumor cells, without affecting its function in normal ones [4,8-10].

Gastric cancer constitutes one of the most common malignant tumors in Asian countries [11,12]. Although its incidence in West countries is lower than those in Asia, it remains a major health problem, representing the second cause of cancer-related deaths worldwide [11,12]. Helicobacter pylori infection and to a lesser extent smoking have been identified as the main environmental risk factors for gastric cancer [13,14]. Recent evidence suggested that HSPs may have a close relationship with gastric neoplasia [15-21]. However, there is little information about their clinical relevance in the management and prognosis of patients with this type of cancer.

The present study aimed to estimate the extent of the immunohistochemical expression of HSP-27, -60 and -90 proteins in tumoral specimens obtained from gastric cancer patients. We also aimed to evaluate the association between the extent and intensity of expression of HSP staining and various clinicopathological parameters, tumor proliferative capacity, and patients' survival.

## 2. Methods

### 2.1. Patients

Sixty-six gastric carcinoma specimens obtained from an equal number of patients who underwent surgical resection due to gastric cancer were included in this study. This study was approved by the ethical committee of Laikon General Hospital. None of the patients received any kind of anti-cancer treatment prior to surgery. Forty-seven of the patients were men (71%) and 19 (29%) women. The mean age of the patient cohort was 67.5 ± 8.6 years (median: 67 years, range: 39–88 years). Tumors were categorized according to Lauren classification [22] as: intestinal type in 30 (45%) and diffuse type in 36 (55%) out of 66 cases. Three levels of differentiation were used to classify grading as: well (n = 3, 5%), moderately (n = 30, 45%) and poorly differentiated (n = 33, 50%). Tumors staging was assessed using the 5^th ^edition of the Tumor, Node, Metastasis (TNM) system according to the Union Internationale Contra la Cancrum (UICC) and the American Joint Committee on Cancer (AJCC) [23]; they were classified asT1 (n = 9, 14%), T2 (n = 22, 33%), T3 (n = 29, 44%) and T4 (2 = 6, 9%). Twenty-four (36%) patients were node negative (N0), and 42 (64%) node positive (N1, n = 38, 58% and N2, n = 4, 6%). Organ metastasis was noted in 7 (11%) out of 66 patients examined. Sixty three patients were followed up for a median of 24 months (mean 28.9 ± 22.3 months, range 1–104 months).

### 2.2 Immunohistochemistry

Immunostainings for HSP-27, -60 and -90 were performed on paraffin-embedded tissue sections using commercially available goat anti-human HSP-27, HSP-60 and HSP-90 (Santa Cruz Biochemicals, Santa Cruz, CA, USA) primary antibodies. Briefly, 4 μm thick tissue sections were dewaxed in xylene and were brought to water through graded alcohols. To remove the endogenous peroxidase activity, sections were then treated with freshly prepared 0.3% hydrogen peroxide in methanol in the dark, for 30 minutes, at room temperature. Non-specific antibody binding was then blocked using Eraser, a specific blocking reagent for goat primary antibodies (Eraser, Biocare Medical, Walnut, Creek, CA, USA) for 8 min. Antigen immunoreactivity was detected by incubation with primary antibodies at a 1:100 dilution, in phosphate buffered saline (PBS) for one hour, at room temperature, according to the manufactures' instructions. After washing three times with PBS, sections were incubated for 30 minutes at room temperature with biotinylated linking reagent donkey, *anti*-goat immunoglobulins diluted 1:150 in PBS (Santa Cruz Biochemicals, Santa Cruz, CA, USA), and rinsed three times with PBS. The sections were then incubated with peroxidase-conjugated streptavidin label (Dakopatts, Glostrup, Denmark), for 20 minutes. The resultant immune peroxidase activity was developed in 0.5% 3,3'-diaminobenzidine hydrochloride (DAB; Sigma, Saint Louis, MO, USA) in PBS containing 0.03% hydrogen peroxide for 3 minutes. Sections were counterstained with Harris' hematoxylin and mounted in Entellan (Merck, Darmstadt, Germany). Appropriate negative controls were performed by omitting the primary antibody and/or substituting it with an irrelevant anti-serum. As positive controls paraffin-embedded liver sections with known immunoreactivity for HSPs were used.

The tumors' proliferative capacity was assessed immunohistochemically, using a mouse *anti*-human Ki-67 antigen; IgG_1k _antibody (clone MIB-1, Dakopatts) as previously described [24]. An additional step of antigen retrieval (citrate buffer at pH 6.1 and microwave heating) was performed before incubation with the primary Ki-67 antibody.

The percentages of positively stained cells were obtained by counting at least 1000 cells in each case by two independent observers (S.T. and S.V.) blinded to the clinical data with complete observer agreement. Specimens were considered "positive" for HSPs when more than 5% of the tumor cells were stained, while they were characterized to present "high expression" for HSPs when the percentage of positively stained cells exceeded the mean percentage value [24]. The intensity of HSP expression was also estimated and graded on a three step scale as: mild (+), moderate (++) and intense (+++) [24].

### 2.3 Statistical Analysis

Chi-square and Fisher's exact tests were used to assess the association between HSP-27, -60 and -90 overexpression and intensity of immunostaining and clinicopathological variables. Spearman rank correlation was used to assess the correlation amongst HSP-27, -60 and -90 protein expressions. Survival curves were constructed using the Kaplan-Meier method and compared using the log-rank test. Cox proportional hazard regression analysis was used to evaluate the effect of HSP-27, -60 and -90 expression and intensity of staining as prognostic factors on patients' survival. A 2-tailed P < 0.05 was considered (statistically) significant. Statistical analyses were performed using the software package SPSS for Windows (version 11.0; SPSS Inc., Chicago, IL, USA).

## 3. Results

**Figure 1:**
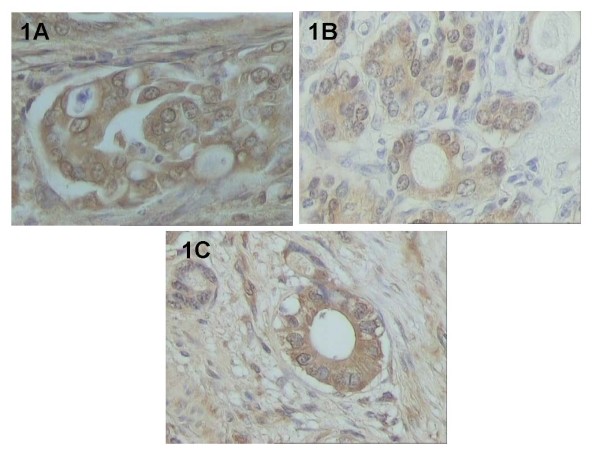
**Representative cases of HSPs protein expression in tumor cells of gastric adenocarcinoma: A. HSP-27 B. HSP-60. C. HSP-90 (original magnification ×200)**.

HSP-27, -60, -90 proteins were abundantly expressed in gastric adenocarcinoma cases examined, presenting mainly a cytoplasmic and occasionally membraneous pattern of staining. Representative immunostainings for HSP-27, -60 and -90 are illustrated in Figure 1A–C.

The mean HSP-27 expression value was 52%, while the incidence of tumors with high HSP-27 expression was 50% (33 out of 66 cases) (Table 1). The intensity of HSP-27 immunostaining was classified as mild in 20 (30%), moderate in 40 (61%) and intense in 6 (9%) cases. In cross-tables, HSP-27 expression was significantly associated with tumor size (pT, P = 0.026), the presence of organ metastases (pM, P = 0.046) and pStage (P = 0.041), while a trend was showed with lymph node positivity (pN, P = 0.078). HSP-27 intensity of staining was significantly associated with lymph node positivity (pN, P = 0.042), while with patients' age was borderline significant (P = 0.070) (Table 1).

**Table 1:** HSP-27 expression and intensity of staining and clinicopathological characteristics of the 66 patients with gastric adenocarcinoma

Clinicopathological Characteristics	HSP-27 expression			HSP-27 intensity			
**n = 66**	**< 52%(%)**	≥ 52% (%)	***p*-value**	**+(%)**	**++(%)**	**+++(%)**	***p*-value**
**All cases**	33(50)	33(50)		20(30)	40(61)	6(9)	
**Age**			0.796				**0.070**
< 67	12(18)	11(17)		10(15)	13(20)	0(0)	
≥ 67	21(32)	22(33)		10(15)	27(41)	6(9)	
**Sex**			0.415				0.892
Male	22(33)	25(38)		15(23)	28(43)	4(6)	
Female	11(17)	8(12)		5(7)	12(18)	2(3)	
**Histological type**			0.460				0.628
Intestinal	15(23)	15(23)		8(12)	20(30)	2(3)	
Diffuse	18(27)	18(27)		12(18)	20(31)	4(6)	
**Histological grade**			0.566				0.266
Well differentiated	1(2)	2(3)		1(2)	2(3)	0(0)	
Moderately differentiated	17(25)	13(20)		7(10)	22(34)	1(2)	
Poorly differentiated	15(23)	18(27)		12(18)	16(24)	5(7)	
**pT classification**			**0.026**				0.661
T1	7(11)	2(3)		5(7)	4(6)	0(0)	
T2	10(15)	12(18)		6(9)	14(21)	2(3)	
T3	16(24)	13(20)		8(12)	18(28)	3(4)	
T4	0(0)	6(9)		1(2)	4(6)	1(2)	
**pN classification**			**0.078**				**0.042**
N0	13(20)	11(17)		6(9)	16(24)	2(3)	
N1	16(24)	22(33)		10(15)	24(37)	4(6)	
N2	4(6)	0(0)		4(6)	0(0)	0(0)	
**pM classification**			**0.046**				0.593
M0	32(48)	27(41)		17(26)	37(57)	5(7)	
M1	1(2)	6(9)		3(4)	3(4)	1(2)	
**pStage**			**0.041**				0.162
I	14(21)	6(9)		8(12)	12(18)	0(0)	
II	7(11)	10(15)		2(3)	11(17)	4(6)	
III	11(17)	19(29)		7(11)	13(20)	1(2)	
IV	1(2)	7(10)		3(4)	4(6)	1(2)	
**Ki-67 protein statement**			0.269				0.865
< 75	26(39)	22(33)		14(21)	30(45)	4(6)	

≥ 75	7(11)	11(17)		6(9)	10(16)	2(3)	

The mean HSP-60 expression value was 52%, while the incidence of tumors with high HSP-60 expression was 55% (36 out of 66 cases) (Table 2). The intensity of HSP-60 immunostaining was classified as mild in 21 (32%), moderate in 32 (48%) and intense in 13 (20%) cases. In cross-tables, HSP-60 expression was significantly associated with patient sex (P = 0.011), while a trend was found with tumor histological grade (P = 0.053) and tumor proliferative capacity (Ki-67 statement, P = 0.077). HSP-60 intensity of staining was significantly associated with patients' age (P = 0.027) and tumor histopathological grade (P = 0.031) (Table 2).

**Table 2:** HSP-60 expression and intensity of staining and clinicopathological characteristics of the 66 patients with gastric adenocarcinoma

Clinicopathological Characteristics	HSP-60 expression			HSP-60 intensity			
**n = 66**	**< 52%(%)**	≥ 52% (%)	***p*-value**	**+(%)**	**++(%)**	**+++(%)**	***p*-value**
**All cases**	30(45)	36(55)		21(32)	32(48)	13(20)	
**Age**			0.187				**0.027**
< 67	13(20)	10(15)		10(15)	6(9)	7(11)	
≥ 67	17(25)	26(40)		11(17)	26(39)	6(9)	
**Sex**			**0.011**				0.436
Male	26(40)	21(32)		17(26)	22(33)	8(12)	
Female	4(5)	15(23)		4(6)	10(15)	5(8)	
**Histological type**			0.191				0.851
Intestinal	11(16)	19(29)		10(15)	15(22)	5(8)	
Diffuse	19(29)	17(26)		11(17)	17(26)	8(12)	
**Histological grade**			**0.053**				**0.031**
Well differentiated	3(4)	0(0)		3(4)	0(0)	0(0)	
Moderately differentiated	10(15)	20(30)		7(11)	19(28)	4(6)	
Poorly differentiated	17(26)	16(25)		11(17)	13(20)	9(14)	
**pT classification**			0.239				0.298
T1	5(7)	4(5)		4(6)	5(8)	0(0)	
T2	8(12)	14(22)		8(12)	10(15)	4(6)	
T3	16(24)	13(21)		9(14)	14(21)	6(10)	
T4	1(2)	5(7)		0(0)	3(4)	3(4)	
**pN classification**			0.250				0.213
N0	14(21)	10(15)		10(15)	11(17)	3(4)	
N1	14(21)	24(37)		9(14)	19(28)	10(16)	
N2	2(3)	2(3)		2(3)	2(3)	0(0)	
**pM classification**			0.884				0.230
M0	27(41)	32(49)		20(30)	29(45)	10(17)	
M1	3(4)	4(6)		1(2)	3(3)	3(3)	
**pStage**			0.114				0.649
I	10(15)	10(15)		8(12)	9(14)	3(4)	
II	4(5)	13(21)		5(7)	10(15)	2(3)	
III	13(21)	8(12)		7(11)	9(14)	5(9)	
IV	3(4)	5(7)		1(2)	4(5)	3(4)	
**Ki-67 protein statement**			**0.077**				0.913
< 75	25(38)	23(34)		15(23)	24(36)	9(14)	

≥ 75	5(7)	13(21)		6(9)	8(12)	4(6)	

The mean HSP-90 expression value was 48%, while the incidence of tumors with high HSP-90 expression was 50% (33 out of 66 cases) (Table 3). The intensity of HSP-90 immunostaining was classified as mild in 30 (45%), moderate in 31 (47%) and intense in 5 (8%) cases. In cross-tables, HSP-90 expression was not significantly associated with any of the clinicopathological parameters examined. HSP-90 intensity of staining was significantly associated with tumor size (pT, P = 0.020). A borderline association (P = 0.070) between HSP-90 intensity of staining and histopathological type was also noted (Table 3).

**Table 3:** HSP-90 expression and intensity of staining and clinicopathological characteristics of the 66 patients with gastric adenocarcinoma

Clinicopathological Characteristics	HSP-90 expression			HSP-90 intensity			
**n = 66**	**< 48%(%)**	≥ 48% (%)	***p*-value**	**+(%)**	**++(%)**	**+++(%)**	***p*-value**
**All cases**	33(50)	33(50)		30(45)	31(47)	5(8)	
**Age**			0.196				0.629
< 67	14(21)	9(14)		12(18)	10(15)	1(2)	
≥ 67	19(29)	24(36)		18(27)	21(32)	4(6)	
**Gender**			0.786				0.276
Male	23(35)	24(36)		22(33)	23(35)	2(3)	
Female	10(15)	9(14)		8(12)	8(12)	3(5)	
**Histological type**			0.621				**0.070**
Intestinal	14(21)	16(24)		13(20)	17(25)	0(0)	
Diffuse	19(29)	17(26)		17(25)	14(22)	5(8)	
**Histological grade**			0.834				0.661
Well differentiated	2(3)	1(2)		2(3)	1(2)	0(0)	
Moderately differentiated	15(22)	15(24)		14(21)	15(22)	1(2)	
Poorly differentiated	17(25)	15(24)		14(21)	15(23)	4(6)	
**pT classification**			0.240				**0.020**
T1	7(11)	2(3)		8(12)	1(2)	0(0)	
T2	9(14)	13(20)		7(10)	14(22)	1(2)	
T3	15(22)	14(21)		13(20)	14(21)	1(2)	
T4	2(3)	4(6)		2(3)	2(3)	2(4)	
**pN classification**			0.873				0.985
N0	13(20)	11(17)		11(17)	11(17)	2(3)	
N1	18(27)	20(30)		17(25)	18(27)	3(5)	
N2	2(3)	2(3)		2(3)	2(3)	0(0)	
**pM classification**			0.689				0.530
M0	29(44)	30(46)		26(40)	29(44)	4(6)	
M1	4(6)	3(4)		4(5)	2(3)	1(2)	
**pStage**			0.250				0.114
I	12(18)	8(12)		10(15)	10(15)	0(0)	
II	5(8)	12(18)		7(10)	7(11)	3(5)	
III	12(18)	9(14)		9(14)	12(18)	0(0)	
IV	4(6)	4(6)		4(6)	2(3)	2(3)	
**Ki-67 protein statement**			1.000				0.260
< 75	24(36)	24(36)		23(35)	20(30)	5(8)	

≥ 75	9(14)	9(14)		7(10)	11(17)	0(0)	

We calculated the Spearman's rank correlation coefficient to evaluate the linear relationships amongst the extent of expression of HSP-27, -60 and -90 proteins (Table 4). Significant positive correlations were obtained between the HSP-27 percentage expression and that of HSP-60 and -90 (r_s _= 0.47, P < 0.001 and r_s _= 0.32, P = 0.008, respectively). A significant positive correlation was also found between the extent of HSP-60 and HSP-90 expressions (r_s _= 0.25, P = 0.044) (Table 4).

**Table 4:** Spearman rank correlations amongst the HSP-27, -60 and -90 proteins extent of expression in the 66 gastric tumoral specimens

n = 66	HSP-27	HSP-60	HSP-90
**HSP-27**	-	r_s _= 0.47, P < 0.001	r_s _= 0.32, P = 0.008
**HSP-60**	r_s _= 0.47, P < 0.001	-	r_s _= 0.25, P = 0.044

**HSP-90**	r_s _= 0.32, P = 0.008	r_s _= 0.25, P = 0.044	-

**Figure 2:**
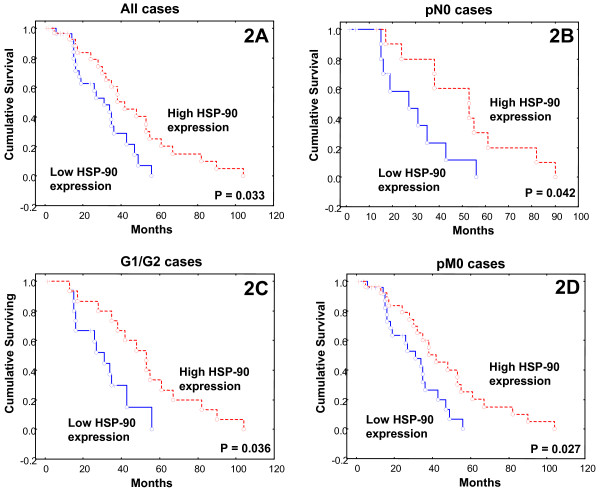
**Kaplan-Meier survival analysis stratified according to HSP-90 expression in patients with gastric cancer**. **A**. All cases. **B**. Subgroup of pN0 cases. **C**. Subgroup of G1/G2 cases. **D**. Subgroup of pM0 cases. (Red dashed line, high HSP-90 expression; Blue continuous line, low HSP-90 expression).

The Kaplan-Meier product-limit method for overall survival analysis according to HSP-27 or -60 expression (low *vs *high HSP-27 or -60 proteins expression) in gastric adenocarcinoma specimens did not reveal significant associations (log-rank test, P = 0.478 or P = 0.953, respectively). Univariate analysis showed significant difference in the survival times of gastric cancer patients with respect to HSP-90 expression (low *vs *high) (log-rank test, P = 0.033) (Figure 2A). Multivariate survival Cox regression analysis also showed statistical significance for HSP-90 expression (P = 0.026). No significant associations between HSP-27, -60 and -90 intensity of immunostaining and patients' survival were noted (P = 0.577, P = 0.094 and P = 0.299, respectively). To further evaluate the association of HSP-27, -60 and -90 intensity of staining with patients' survival, we grouped carcinomas which had moderate or intense intensity of staining together and opposed them to cases with mild intensity. In this context, no significant associations were noted for HSP-27, -60 and -90 immunostainings (P = 0.965, P = 0.551 and P = 0.072, respectively).

To assess if HSPs bear a pronounced prognostic effect in patient subgroups, we conducted an extensive Kaplan-Meier analysis of HSP-27, -60 and -90 protein expressions (low *vs *high) and intensity of staining (mild *vs *moderate and intense). We stratified by pT stage (small *vs *large tumor size, pT1/T2 *vs *pT3/T4), tumor grading (poorly *vs *moderately and well differentiated), nodal status (absence *vs *presence of lymph node metastases, pN0 *vs *pN1/N2), presence of organ metastases (pM0 *vs *pM1) and histopathological type (intestinal *vs *diffuse). Stratifying for histological type, HSP-90 expression and intensity of immunostaining were associated with longer overall survival times in intestinal type gastric cancer cases, without though reaching statistical significance (log-rank test, P = 0.069, P = 0.064, respectively). Such an association was not seen in diffuse type (log-rank test, P = 0.373) (data not shown). In the subgroup of lymph node negative gastric cancer cases (pN0), high HSP-90 expression was significantly associated with longer survival times (P = 0.042) (Figure 2B); however, this association did not remain in the multivariate analysis (P = 0.111). In the subgroup of well and moderately differentiated (G1/G2) gastric cancer cases, high HSP-90 expression was significantly associated with longer overall survival times (P = 0.036) (Figure 2C), being also identified as an independent prognostic factor in multivariate analysis (P = 0.018), which was not seen in poorly differentiated (G0) gastric cancer cases (P = 0.754). Stratifying for organ metastasis, in negative cancer cases (pM0), high HSP-90 expression was significantly associated with longer overall survival time (P = 0.027) (Figure 2D), being also identified as an independent prognostic factor in multivariate analysis (P = 0.032). No significant associations of HSP-27, -60 and -90 expression or intensity of immunostaining with patients' survival were noted in all the other examined subgroups of patients (data not shown).

## 4. Discussion

HSPs have been reported to be overexpressed in a wide range of human tumors, while HSPs expression has been associated with tumor cell growth and differentiation, as well as with resistance to apoptosis and poor prognosis [7]. There is also relatively recent evidence to support that HSPs are overexpressed in human gastric cancer and may contribute to the development and prognosis of this type of cancer [7].

The present study further supports the clinical significance of HSP-27, -60 and -90 protein expression in the progression of gastric cancer. All examined cases of gastric adenocarcinoma were tested positive for HSP-27, -60 and -90. This incidence of HSP-27, -60 and -90, positivity is among the highest reported for this specific type of tumor malignancy. Both Takano et al. and Kapranos et al. reported a lower incidence of HSP-27 positivity: 52.4% (22/42) and 62.7% (54/86), respectively [20,21]. In addition, concerning HSP-70 and HSP-40, two other members of HSPs family, positive staining was noted at a percentage of 67.9% (55/81) and 22.2% (18/81) of gastric cancer cases [15]. However, Zuo et al. reported results similar to ours with HSP-90α positivity of 95% (84/88) [18].

The present study further showed that HSP-27, -60 and -90 were highly expressed in gastric cancer tissues with mean percentage expression values of 52, 52 and 48%, respectively. Overexpression concerning a group of HSPs, including HSP-60, HSP-70 and Chaperonin containing TCP1 (CCT), a member of HSP-60 family, in a rather low sample of 10 primary gastric adenocarcinoma cases was reported by proteomic analysis. The authors suggested that this overexpression may be attributed to the stress response and self-protective effort of the cells during malignant transformation [17]. However, the exact molecular mechanisms through which HSPs become overexpressed in cancer remain to be clarified. Overall, the available data so far suggest that HSPs may well be related to the genetic changes implicated in tumor progression. Oncoproteins may appear during carcinogenesis, such as mutated p53, and these mutated and conformationally altered proteins may elicit an HSP response [1].

Significant positive correlations (Spearmen rank correlation) were found between the extent of HSP-27 expression and that of HSP-60 and -90 proteins (Table 4). A lower correlation (Spearmen rank correlation) between the expression of HSP-60 and that of HSP-90 was also obtained (Table 4). However, in another study involving oesophageal squamous cell carcinoma, no correlation between HSP-60 and HSP-90 was reported [25].

In the present study, HSP-27 expression and intensity of immunostaining were found to be associated with important clinicopathological characteristics for patients' management. This result is in line with previous evidence that HSP-27 expression was reported to be associated with lymph node metastases in 86 human gastric carcinoma samples [21]. Consistently, in our study, significant and borderline associations between HSP-27 intensity and expression of immunostaining and nodal status were found, respectively. In addition, HSP-27 expression was significantly associated with the presence of organ metastasis, tumor size and pStage. However, HSP-27 expression and staining intensity were not associated with patients' survival. In this context, Kapranos et al. demonstrated that HSP-27 expression in 86 gastric cancer specimens was associated with shorter overall survival in univariate analysis; however, this relationship was not remained in multivariate analysis [21]. Moreover, according to Takano et al., univariate analysis of 42 tissue samples from patients with resectable stage IV gastric cancer showed that pN factors, blood vessel invasion, HSP-27 overexpression and the index of p53 and HSP-27 overexpression exhibited a prognostic influence [20]. However, only the Lauren classification was sustained as an independent variable in the multivariate analysis [20]. Furthermore, we found that patients with diffuse type of gastric cancer were characterized by shorter survival times when compared with those with intestinal type; however, this difference did not reach statistical significance (log-rank test, p = 0.083, data not shown). Both Maehara et al. and Isomoto et al. also did not find any significant association between HSPs expression and patient survival [15,16]. In fact, histopathological differentiation was significantly correlated with the expression of HSP-70, whereas no impact of HSP-70 and HSP-40 expression on any factors predicting prognosis and survival was noted [15,16]. Another study by Wang et al. further revealed a significant correlation between the expression of HSP-72 and gp96 and the progression of gastric carcinomas [26].

HSP-60 expression was significantly associated with patient sex, with only borderline associations with tumor histopathological grade and proliferative capacity reflected by Ki-67 labelling index. In fact, higher HSP-60 expression was found in female compared to male patients, while poorly differentiated tumors more frequently expressed higher levels of HSP-60 compared to well and moderate differentiated ones. In addition, HSP-60 staining intensity was significantly associated with patients' age and tumor histopathological grade. Accordingly, Cappello et al. showed that HSP-60 expression was associated with tumor grade in advanced large bowel carcinomas with lymph node metastases [27]. HSP-60 was also reported to be overexpressed early in prostate and colorectal carcinogenesis [28,29]. Moreover, it was supported that HSP-60 may be a novel biomarker during bronchial carcinogenesis [30]. On the other hand, although HSP-60 expression was not associated with any clinicopathological parameters in oesophageal squamous cell carcinoma patients, it was significantly associated with the apoptotic index and patient prognosis [25]. In contrast, we did not find any significant association of HSP-60 expression or staining intensity with the prognosis of patients with gastric cancer. To our knowledge, prior to the current study, there was no available data concerning the clinical significance of HSP-60 expression in relation to clinicopathological parameters in gastric cancer.

According to our results, HSP-90 protein expression was not associated with any of the examined clinicopathological characteristics. However, HSP-90 intensity of immunostaining was significantly associated with tumor size, and borderline with histological type. Overall, the diagnostic value of HSP-90 expression has been examined in several types of cancers; however, most of the studies so far did not find significant associations with clinicopathological characteristics [7]. With respect to gastric cancer, a significant association was reported by Zuo et al. between HSP-90α expression and lymph node metastases [18]. In addition, enhanced HSP-90β expression was also demonstrated in human gastric cancer tissues and especially in poorly differentiated types [19]. *In vitro *studies also showed that HSP-90β expression was greater in SGC7901/VCR of MDR-type gastric cell line than in its parental cell line SGC7901 [19].

To our knowledge, there is no available evidence so far concerning the prognostic value of HSP-90 expression in gastric cancer. In this respect, the current study is the first report examining the clinical significance of HSP-90 expression in the prognosis of patients with gastric adenocarcinoma. On univariate survival analysis, higher HSP-90 protein expression was associated with longer overall survival times. Moreover, the multivariate survival analysis showed that HSP-90 protein expression was a significant independent prognostic factor for gastric adenocarcinoma patients. In the clinically distinctive subgroups of node-negative, well and moderately differentiated and organ metastasis-negative tumors, high HSP-90 protein expression was also associated with favorable prognosis. Moreover, HSP-90 expression and intensity of immunostaining showed a trend to be associated with overall survival times in intestinal type, but not in diffuse type gastric cancer cases. The contradictory prognostic value of HSP-90 overexpression between diffuse and intestinal type of gastric cancer patients could be attributed to the different signals produced from the tumor microenvironment and the individual cellular characteristics of each tumor histological type. Such distinct characteristics could trigger tumoral cells to upregulate or downregulate HSP-90 signalling in respect to tumor histological type. The prognostic value of HSP-90 expression has also been examined in several types of cancer. In agreement with our results, HSP-90 expression was significantly associated with favorable prognosis in endometrial cancer [31,32]. In contrast, HSP-90 expression in cancer tissues and the presence of autoantibodies to HSP-90 have been associated with poor prognosis in breast cancer [33], while it was found to be of no prognostic value in ovarian and oral carcinomas [25,34,35]. The present data that HSP-90 expression is associated with improved survival deserves special attention, as it could be implied that loss of chaperoning, such as HSP-90, may lead to a more aggressive phenotype, thus leading to poor prognosis or resistance to therapy. In this context, blocking HSP-90 was shown to disrupt multiple proangiogenic signaling pathways in gastric cancer cells and inhibit xenografted tumor growth *in vivo *[36]. Hence, gastric cancer harbors attractive molecular targets for therapy with HSP-90 inhibitors, which could lead to improved efficacy of antineoplastic therapy regimens [36].

## 5. Conclusion

The current study suggested that the expression of HSP-27, -60 and -90 proteins was associated with important clinicopathological parameters in respect to the diagnosis of patients with gastric cancer. HSP-27 and -60 failed to predict patients' prognosis, whereas HSP-90 was shown to be an independent prognostic indicator in patients with gastric cancer. Further research is required in order to clarify the mechanisms through which HSPs' regulation affects the molecular events involved in tumor growth, invasiveness and metastases. Such studies could be essential for the development of new therapeutic strategies, such as HSP-90 inhibitors, and promising prognostic indicators in gastric cancer. It was also suggested that depending on their mode of induction, intracellular/extracellular location, cellular origin (eukaryote/prokaryote), peptide loading status, intracellular ADP/ATP content, concentration, and route of application, HSPs may either exert immune activation as danger signals in cancer immunity and mediate protection against infectious diseases or exhibit regulatory activities in controlling and preventing autoimmunity [37]. In this context, further studies should be conducted on measuring serum HSPs levels in order to delineate whether there is a link between intracellular and extracellular HSPs levels in gastric neoplasia.

## Competing interests

The authors declare that they have no competing interests.

## Authors' contributions

CG carried out the immunostainings, drafted the paper, SSD performed the statistical analysis, SV carried out the immunostainings and the immunohistochemistry data evaluation, IS contributed to the histopathology and clinical data collection, GK participated in the design and coordination of the study, SET participated in the design of the study, carried out the immunohistochemistry data evaluation and revised the manuscript. All authors read and approved the final manuscript.

## Pre-publication history

The pre-publication history for this paper can be accessed here:

http://www.biomedcentral.com/1471-230X/9/14/prepub
